# Youth Reproductive Health Service Utilization and Associated Factors among Amhara Region Female Night Students, Ethiopia

**DOI:** 10.1155/2021/6640219

**Published:** 2021-04-07

**Authors:** Tewachew Muche Liyeh, Yitayal Ayalew Goshu, Habtamu Gebrehana Belay, Habtamu Abie Tasew, Gedefaye Nibret Mihiretie, Abeba Belay Ayalew

**Affiliations:** Department of Midwifery, College of Health Sciences, Debre Tabor University, Ethiopia

## Abstract

**Introduction:**

Youth is a decisive age to shape the direction of their life and that of their family. However, due to the host of biological, social, and economic factors, adolescent females can be at high risk of adverse sexual and reproductive health outcomes. Therefore, assessing youth reproductive service utilization and associated factors among female night students is very crucial for timely intervention to their gaps.

**Method:**

An institutional-based cross-sectional study design was conducted in Amhara region among 2,050 female night students from September 15 to November 15, 2018. A self-administered pretested questionnaire was used to collect the data. Bivariate and multivariable logistic regression models were used. Odds ratio with 95% confidence interval was computed to determine the strength of association between predictor and outcome variables. *P* value less than or equal to 0.05 is considered as the level of significance.

**Results:**

Out of the total respondents, about 54.6%(CI: 52.5%-56.8%) of them utilized reproductive health services. Respondents who were attending secondary education (AOR = 2.55, 95%CI = 1.97‐5.62), attitude towards youth reproductive health services (AOR = 2.74, 95%CI = 2.07‐5.30), those who had a habit of communicating on sexual and reproductive health issues with their family (AOR = 3.66, 95%CI = 3.59‐7.41), discussion on sexual and reproductive health issue with peers/friends (AOR = 1.43, 95%CI = 1.01‐2.02), respondents with good knowledge on youth reproductive health services (AOR = 2.03, 95%CI = 1.49‐2.75), and those who had faced reproductive health problems (AOR = 2.03, 95%CI = 1.49‐2.75) were significantly associated with youth reproductive health service utilization.

**Conclusion:**

Youth reproductive health service utilization among female night students was not satisfactory. Therefore, special focus should be given to female night students by providing accessible, acceptable, confidential, flexible, and friendly reproductive health service utilization. Finally, community health promotion and education are mandatory to promote the practice of discussing youth reproductive health issues with their children.

## 1. Introduction

Youth is a period of transition from childhood to adulthood characterized by significant physiological, psychological, and social changes that place their life at high risk [1]. According to the World Health Organization (WHO), youth is defined as a young person between the age group of 15 and 24 years [2]. More than a quarter of the world's population is between the ages of 10 and 24, with 86% living in less developed countries [3]. More than 1/3 of the Ethiopian population is found between 10 and 24 years in which they are the most vulnerable to a range of sexual and reproductive health problems [4].

Young people can be labeled as the vulnerable group, because this segment of population is subjected to curiosity, sexual maturity, and natural inclination towards experimentation, and peer pressure leads to risky behavior [5]. Many girls in developing countries are vulnerable to leaving school, child marriage, early pregnancy, human immunodeficiency virus (HIV), sexual exploitation, coercion and violence, unwanted pregnancy, and unsafe abortion and its complications [6].

The need to have a healthy youth is of great value to the nation's socioeconomic development. The reproductive and sexual health decisions they make today will affect the health and wellbeing of their communities and of their countries for the future. After the 1994 international conference on population and development in Cairo, many countries have started giving an emphasis to the problems of youth and adolescents [7].

To solve the problems of youth, our country Ethiopia establishes youth reproductive health services (YRHS) for addressing the reproductive and sexual health needs of youth. In Ethiopia, the YSRH services are provided in the already existing health facilities by specially trained health care providers together with peer educators in a separate corner having waiting and consultation spaces. The service packages include counseling and testing for HIV and other sexually transmitted infections, pregnancy test, counseling for contraceptives and offering full range of contraceptives, antenatal care, postnatal care, postabortion care, referral service for antiretroviral therapy, delivery and prevention of mother-to-child transmission of HIV, and promotion of condom. However, youths often lack basic reproductive health information, knowledge, and access to affordable and confidential health services for reproductive health [8, 9].

Many of them do not feel comfortable discussing reproductive health (RH) issues with their parent. Likewise, parents, health care workers, and educators frequently are unwilling or unable to provide complete, accurate, age-appropriate RH information to young people. This is often due to parents' discomfort about the subject or the false belief that providing the information will encourage sexual activity [9].

Night school students in Ethiopia are students who are unable to continue their day time education due to resource limitations, absence of supporters, and working conditions. In our context, most of the female night school students are housemaids and daily laborers. Therefore, they are supposed to have limited awareness about sexual and reproductive health services which may affect their utilization [10].

Even though few research works have been done on daytime students, there is no study conducted concerning youth reproductive health service utilization focusing on adolescent girls attending night school in Ethiopia generally and in Amhara region particularly. Therefore, assessing youth reproductive health service utilization among female night students and identifying factors affecting their utilization are very vital in designing, implementing, and monitoring effective youth reproductive health intervention programs for these particular groups.

## 2. Methods and Materials

### 2.1. Study Design and Setting

A school-based cross-sectional study using a quantitative method of data collection was conducted from September 15, 2018, to November 15, 2018, in Amhara region, northwest Ethiopia. Amhara region is one of the nine regional states in Ethiopia which is found between 11°30′00^″^N latitude and 38°30′00^″^E longitude on the northwestern part of Ethiopia. Amhara is one of Ethiopia's largest regions; it has 12 zones, three city administrations, and 180 woredas (139 rural and 41 urban). According to the Ethiopian Central Statistics Agency, the region has a projected population of 21.5 million people, about 80 percent of whom are rural farmers. The region has 80 hospitals (5 referral, 2 general, and 73 primaries), 847 health centers, and 3,342 health posts. There are 55 elementary and 23 secondary schools that provide night education.

### 2.2. Participants

All female night students aged 15-24 years in Amhara region were the source population of the study. All youth female night students attending elementary and secondary school in selected zones of Amhara region during the study period were considered as the study population.

### 2.3. Sample Size Determination

The sample size was calculated using single population proportion formula by taking proportion of youth reproductive health utilization (32%), 95% confidence interval, 3% margin of error, design effect of 2, and 10% of nonresponse rate. Finally, a total 2,050 participants were included for the study.

### 2.4. Sampling Procedure

A multistage sampling technique was used to select the study participants. In the first stage, 5 zones were selected by using a simple random sampling technique from a total of 12 zones within the region. In the second stage, 22 night schools were selected by lottery method from 88 night schools in the selected zones and the sample size was proportionally allocated to all selected elementary and secondary schools. Finally, study units were selected by using a simple random sampling technique.

### 2.5. Operational Definitions

- Youth: a person with an age group of 15–24-year-old
- Knowledge on YRHS: If the respondents mentioned at least five YRHS on their own, they were considered to have good knowledge; otherwise, they were considered to have poor knowledge
- Attitude of the respondents on YRHS: we assessed the attitude of the respondents regarding YRHS using a five-point Likert scale. The scoring system used was as follows: strongly disagree = 1, disagree = 2, indifferent = 3, agree = 4, and strongly agree = 5. Then, the responses were calculated and those respondents who scored the mean score and above were considered as having a positive attitude, whereas those respondents who scored below the mean score were categorized as negative attitudes towards YRHS
- Youth reproductive health service utilization: those respondents who utilize at least one of the following main RH services in the past one year (pregnancy test, ANC, delivery service, PNC, VCT, FP, STI diagnosis and treatment, abortion and postabortion care, get information, and counselling on sexuality)
- Reproductive health problems: those respondents who faced at least one of RH problems (unwanted pregnancy, abortion, sexual violence, teenage pregnancy, and STI)

### 2.6. Data Collection Instruments and Data Collection Procedure

The data was collected by interviewer-administered, semistructured questionnaires adapted from different literature [11–15]. The data collection tool (questionnaire) was first prepared in English and then translated to a local language (Amharic) and then retranslated back to English language by language experts. The questionnaire comprises socioeconomic and demographic characteristics, individual level, and communication-related factors. About 20 data collectors and 10 supervisors were involved during data collection.

### 2.7. Data Quality Assurance

To assure the quality of the data, technical training was given for data collectors and supervisors for three days. Pretest was given for 5% of the sample size in out of selected schools. During data collection, supervision was conducted. After data collection, checking of data entry and cleaning were conducted for the completeness of the data. Throughout the course of the data collection, interviewers were supervised at each site, and regular meetings were held between the data collectors, supervisor, and the principal investigator together in which problematic issues arising from interviews were discussed and addressed. The collected data were reviewed and checked for completeness before data entry; the incomplete data was discarded.

### 2.8. Data Analysis

The data were entered into EPI data statistical software and then sorted, cleaned, and analyzed by using the SPSS version 20 statistical package. Descriptive statistics were done to describe the study population in relation to relevant variables by using text, tables, and graphs. Bivariate and multivariable logistic regression models were used. Odds ratio with 95% confidence interval was computed to determine the strength of association between predictor and outcome variables. *P* value less than or equal to 0.05 is considered as the level of significance.

### 2.9. Ethical Approval and Consent to Participate

Ethical clearance for this study was obtained from the ethical review board of Debre Tabor University College of Health Sciences, and a supporting letter was obtained from Amhara Regional Education Bureau. This support letter was sought to each zonal town and forwards to elementary and secondary school on which the study was conducted. Informed consent for ≥18 years old and assent for <18 years from their family were obtained after explaining the purpose and objective of the study. An interview was taken place in a convenient place to maintain privacy and to assure confidentiality.

## 3. Results

From a total of 2,050 students included in the study, about 2,044 of them responded to the questions correctly yielding a response rate of 99.7%.

### 3.1. Sociodemographic Characteristics of Respondents

The mean (±) age of the respondents was 20 ± 2.56 years old, and 1,199 (58.7%) of the respondents were within the age group of 15-19 years. More than two-thirds of respondents 1,458 (71.3%) were attending elementary education (1-8 grade). Majority of respondents 1,625 (79.5%) were orthodox religion follower, and 1,906 (93.2%) were Amhara in ethnicity. Almost two-thirds of childhood residents 1,266 (64.6%) were from a rural area. Nearly one-third of respondents were living with an employer and relative 650 (31.8%) (Table 1).

### 3.2. Knowledge of Respondents about Youth Reproductive Health Services

Two-thirds 1,354 (66.24%) of youths heard about the YRS issue. The common source of information was mass media (65%) and school (49.2%). Mentioned components of SRH service by respondents were family planning 1,271 (62.2%) and voluntary counseling and testing 999 (48.9%). About 1,233 (60.3%) of the respondents had good knowledge on YRHS (Table 2).

### 3.3. Reproductive Health Problems of the Respondents

In this study, the reproductive health problems of the students were assessed. Nearly half 1,040 (50.8%) of the students had faced reproductive health problems considering that students faced at least one of reproductive health problems (unwanted pregnancy, abortion, sexual violence, teenage pregnancy, and STI). Out of the 2,044 respondents, 464 (22.7%) had unwanted pregnancy and 986 (42.4%) had faced sexual violence (Table 3).

### 3.4. Preference of Youth Reproductive Health Services among Respondents

Preference of the students in terms of health institution, time of provision, and service provider regarding youth reproductive health services was assessed. About 864 (42.27%) and 112 (5.5%) of respondents reported that they thought better place for provision of SRH services was at public health institutions and schools, respectively. While assessing the time preference of students for RH services, 892 (43.6%) of respondents preferred service time to be in the absence of other users and 1,126 (55%) preferred service provider to be young and of the same sex (Table 4).

### 3.5. Attitudes of the Respondents towards YRHS

We assessed the attitude of the respondents regarding YRHS using the Likert scale. The responses were summed, and the mean score was obtained. Those respondents who scored the mean value and above were considered as having a positive attitude, whereas those respondents who scored below the mean value were categorized as negative attitudes towards YRHS. In this study, out of the total respondents, 1,138 (55.7%) had a positive attitude towards youth reproductive health utilization.

### 3.6. Youth Reproductive Health Service Utilization

**Figure 1:**
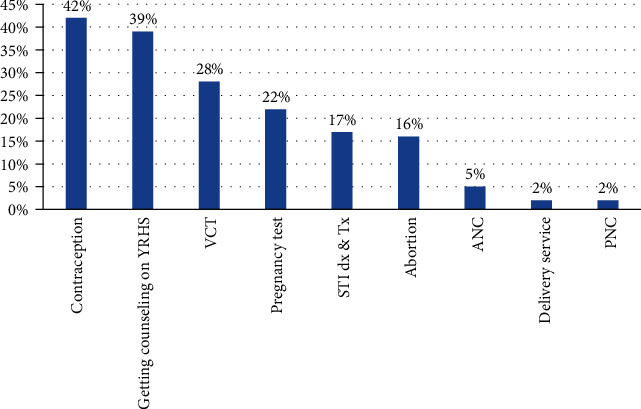
Reproductive health service utilization among female night students in Amhara region, Ethiopia, 2018 (*n* = 1,117).

More than half of the participants 1,117 (54.6%) were utilized at least one youth reproductive health service in the last 12 months. Of whom, 464 (42%) used contraceptives, 313 (28%) got voluntary counseling and testing HIV/AIDS, and 435 (39%) got information, education, and communication about SRH issue (Figure 1).

### 3.7. Reasons for Not Utilizing YRH Services

**Figure 2:**
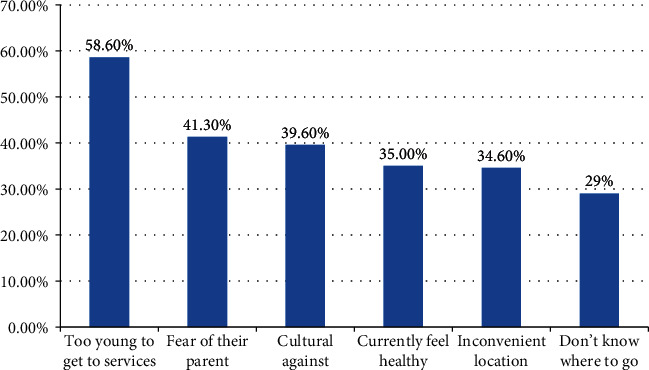
Reasons for not utilizing YRH services among female night students in Amhara region, Ethiopia, 2018 (*n* = 927).

Of the total respondents, 927 (45.4%) of them were not utilized at least one youth reproductive health service. The main reason for not seeking the service was too young to the services (58.6%), afraid of their parent (41.3%), and being embarrassed from cultural against to get the service (39.6%) (Figure 2).

### 3.8. Factors Associated with Utilization of Youth Reproductive Health Services

Respondents who were attending secondary education were 2.6 times more likely to utilize YRH service as compared with those who were attending elementary education (AOR = 2.55, 95%CI = 1.97‐5.62). Students who had a positive attitude towards YRS were 2.7 times more likely to utilize YRH services than those who had a negative attitude (AOR = 2.74, 95%CI = 3.57‐6.3). Respondents who had a habit of communicating with their family about SRH issue were 3.66 times more likely to utilize SRH service than those who had not (AOR = 3.66, 95%CI = 2.59‐7.41). Students who had a history of SRH problems were 7.17 times more likely to utilize YRH services than those who had not (AOR = 7.17, 95%CI = 5.25‐9.79). Participants who had good knowledge on YRS were 2 times more likely to utilize YRH services than those who had poor knowledge (AOR = 2.03, 95%CI = 1.49‐2.75). Respondents who had a habit of communicating with peers about SRH issue were 1.4 times more likely to utilize SRH service than those who had not (AOR = 1.43, 95%CI = 1.01‐2.02) (Table 5).

## 4. Discussion

Young people have limited access to reproductive health service utilization that focuses on the special needs of adolescents. Inadequate knowledge about adolescent sexual behavior, cultural influences, and the limited capacity of implementers hinders the provision of reproductive health education and services to young people. Hence, this study was aimed at assessing the YRHS utilization and associated factors among Amhara region female night students in Ethiopia. It has tried to include youths aged between 15 and 24 years who were residing in the study area.

In this study, only 54.6% of the students were utilized youth reproductive health services. Educational status of respondents, attitude towards YRHS utilization, ever faced reproductive problems, habit of communicating with family about SRH issue, discussion with peers on sexual and reproductive health issues, and knowledge of respondents on YRHS were significantly associated with utilization of YRH service.

In this study, 54.6% (95%CI = 52.5%‐56.8%) of the students were utilized youth reproductive health services within the last one year. This finding is in line with the study done in North Shewa, Amhara region 54.7% [11], but higher than studies conducted in Bahir Dar 32% [12], Woreta town, South Gondar zone 24.6% [13], Nekemte 21.2% [14], and Metekel 33% [15]. This finding is lower than the study done in Mekelle town, northern Ethiopia 69.1% [16] and Harar town, east Ethiopia 63.8% [17]. This difference might be due to the participant's sociodemographic and socioeconomic characteristics, target population difference, definition of YRH service utilization, and availability and accessibility of SRH service.

Commonly utilized components of YRH services were modern contraception, 42% of which is a similar finding in North Shewa (40%) and Mecha district, northwest Ethiopia (35.5%) [11, 18].

Students who were attending secondary education were 2.6 times more likely to utilize YRH service than those who were attending elementary education. This finding is supported by a study conducted in Woreta town, South Gondar zone [13]. The possible resean might be when students were more educated they might have better understanding on sexual and reproductive health issues which increases their health care-seeking behavior

Students who had a positive attitude towards YRHS were 2.7 times more likely to utilize YRH services than those who had a negative attitude. This is due to the fact that students having a favorable attitude would motivate them to use reproductive service utilization.

Respondents who had a habit of communicating with family about SRH issue were 3.66 times more likely to utilize SRH service than those who had not. This is supported by similar findings from Mecha district, Gondar, East Gojjam zone, and Kenya [18–21]. This might be due to the fact that open discussion about SRH issue between family and their daughter increases awareness and avoids feeling shy and fear of being seen while getting SRH service. Moreover, the discussion creates more opportunities to exchange SRH information and experience of health-related problems and influence utilization of the YRH service.

Respondents who had a habit of communicating with peers about SRH issue were 1.4 times more likely to utilize YRH service than those who had not. This finding was supported by a study conducted at Madawalabu University [22]. This might be explained by students who had free discussion with their friends regarding sexual issues; they would have better knowledge on SRH issues which in turn leads to utilization of the service.

Participants who had good knowledge on YRS were 2 times more likely to utilize YRH services than those who had poor knowledge. This finding is in line with the study done in Mekelle and East Gojjam [15, 19]. This might be due to the fact that students with better knowledge and understanding about sexual and reproductive health issues might have better decision-making skills for reproductive health service utilization.

Students who had a history of SRH problems were 7.17 times more likely to utilize YRH services than those who had not. This finding was supported by the study done at Bahir Dar [12]. This might be explained by students who faced reproductive problems like unwanted pregnancy, sexual violence, or abortion; they are more likely to visit health facilities to get youth reproductive health service utilization.

### 4.1. Strength and Limitation of the Study

Using a large sample size can be taken as the strength of the study. Even though the study has strengths, it has its own limitations: the first limitation would be due to sensitivity of the issues, social desirability bias might be faced. Another limitation would be it might be subjected to recall bias since respondents were requested to answer their past experiences.

## 5. Conclusion and Recommendation

Youth reproductive health service utilization in the study area was not satisfactory. Inconvenient location, inconvenient service time, cultural barriers, lack of information on where to get the service, fear of their parent, and perceived as too young to get the services were the main reasons for not utilizing YRS services by female students. Educational status, habit of discussion on sexual and reproductive health issues with family, attitudes towards YRS, knowledge on RH services, ever faced RH problems, and ever had a discussion with peers about sexual and reproductive health issues were factors found to be associated with youth reproductive health service utilization.

Therefore, special focus should be given for female night students by providing accessible, acceptable, confidential, and friendly reproductive health services. Youth reproductive health services shall be given in a convenient place at a time when there are less people around and with a convenient time such as after school, evening, and/or weekend hours. Furthermore, government and other stakeholders should strengthen youth awareness creation strategies and empower them to have positive attitudes towards using YRS services. Finally, community health promotion and education are mandatory to promote the practice of discussing youth reproductive health issues with their children.

## Figures and Tables

**Table 1:** Sociodemographic characteristic of female night students in Amhara region, Ethiopia, 2018.

Variables (*n* = 2,044)	Frequency (*n*)	Percentage (%)
Age		
15-19	1,199	58.7
20-24	845	41.3
Grade level		
Elementary school (grades 1-8)	1,458	71.3
Secondary school (grades 9-10)	586	28.7
Religion		
Orthodox	1,625	79.5
Muslim	318	15.6
Others^∗^	101	4.9
Ethnicity		
Amhara	1,906	93.2
Others^∗∗^	138	6.8
Childhood resident		
Urban	778	38.1
Rural	1,266	64.6
Marital status		
Married	359	17.6
Had boyfriend	892	43.6
Had no partner	793	38.8
Maternal education		
No formal education	1,111	54.4
Primary educational level	595	29.1
Secondary education and above	338	16.5
Living arrangement of the respondent		
With family	408	20
With partner	249	12.2
With friends	387	18.9
With employer	650	31.8
Alone	350	17.1
Pocket money		
No money	557	27.3
<500 ETB	390	19
≥500 ETB	1,097	53.7

∗ = protestant, catholic; ^∗∗^ = Tigre and Oromo; 1 USD = 38 ETB.

**Table 2:** Knowledge about youth reproductive health services among female night students in Amhara region, 2018.

Variable (*n* = 2,044)	Frequency	Percentage
Have ever heard about YRHS?		
Yes	1,354	66.24
No	690	33.76
From where you got information about youth YRHS service? (multiple responses)		
Family	549	49.2
Relatives	367	27
School	612	49.2
Heath institution	421	31
Friend/peers	795	58.7
Mass media	882	65
Which types of services you know were provided under a youth-friendly clinic? (multiple responses)		
Family planning	1,271	62.2
VCT	999	48.9
Treatment for STI	581	28.4
Sexual education	511	25
Pregnancy testing	625	30.6
Abortion service	961	47
Postabortion care services	713	34.9
Antenatal care	608	29.8
Postnatal care	543	26.6
Counseling about SRH issues	468	22.9
Delivery service	928	45.4

**Table 3:** Reproductive health problems among female night students in Amhara region, Ethiopia, 2018.

Variable (*n* = 2,044)	Frequency (*n*)	Percentage (%)
How old were you during your first pregnancy? (*n* = 359)		
<18 years	234	39
>18 years	366	61
Have you ever faced unwanted pregnancy?		
Yes	464	22.7
No	1,580	77.3
Have you ever faced unsafe abortion?		
Yes	136	6.65
No	1,908	93.35
Have you ever faced STI?		
Yes	98	4.8
No	1,946	95.2
Have you ever faced sexual violence?		
Yes	986	48.24
No	1,058	51.76

**Table 4:** Preference of youth reproductive health services among female night students in Amhara region, Ethiopia, 2018.

Variable (*n* = 2,044)	Frequency	Percentage
Institutions preferred for RH service		
Government health institute	864	42.27
Government youth clinics	694	34
Private health institute	213	10.3
FGAE clinics	159	7.8
School	112	5.5
Convenient time for YRH service		
In the usual health institute working hours	790	38.7
In the hours when other users are not around	892	43.6
The service shall be given for 24 hours	362	17.7
Preferred provider for YRH		
Young provider of the same sex	1,126	55
Young provider of any sex	287	14
Adult provider of the same sex	469	23
Any provider could be	162	8

**Table 5:** Multivariable logistic regressions on factors associated with utilization of youth RH services among female night school students, Amhara region, Ethiopia, 2018.

Variables (*n* = 2,044)	RH service utilization		COR (95% CI)	AOR (95% CI)	*P* value
	Yes	No			
Educational status					
Elementary school (grades1-8)	508	696	1	1	
Secondary school (grades 9-10)	609	231	3.6 (2.99-4.37)	2.55 (1.97-5.62)^∗^	0.0001
Attitude for SRH service					
Favorable attitude	760	378	3.09 (2.578-3.71)	2.74 (3.57-6.3)^∗^	0.0001
Unfavorable attitude	357	549	1	1	
Habit of communicating about SRH issues with family					
Yes	830	371	4.33 (3.59-5.23)	3.66 (2.59-7.41)^∗^	0.0001
No	287	556	1	1	
Ever faced SRH problem					
Yes	818	222	8.69 (7.11-10.62)	7.17 (5.25-9.79)^∗^	0.0001
No	299	705	1	1	
Discussion with peers on SRH					
Yes	422	137	3.50 (1.28-4.35)	1.43 (1.01-2.02)^∗^	0.045
No	695	790	1		
Knowledge on YRH services					
Good	891	342	6.74 (5.53-8.22)	2.03 (1.49-2.75)^∗^	
Poor	226	585	1	1	0.001
Ever had sexual intercourse					
Yes	827	375	4.20 (3.48-5.06)	0.53 (0.25-1.11)	0.095
No	290	552	1	1	

∗ = significantly associated variables.

## Data Availability

All relevant data are available within the manuscript.

## Abbreviations

CI: Confidence interval
IEC: Information, education, and communication
RH: Reproductive health
RHS: Reproductive health service
SRHS: Sexual and reproductive health service
VCT: Voluntary counseling and testing
WHO: World Health Organization
YRHS: Youth reproductive health service.
